# Compensation for Matrix Effects in High-Dimensional Spectral Data Using Standard Addition

**DOI:** 10.3390/s25030612

**Published:** 2025-01-21

**Authors:** Elena Khanonkin, Israel Schechter, Itai Dattner

**Affiliations:** 1 Faculty of Chemistry, Technion-Israel Institute of Technology, Haifa 32000, Israel; 2 Department of Statistics, University of Haifa, 199 Abba Khoushy, Haifa 3498838, Israel

**Keywords:** high dimensions, matrix effect, principal component regression, standard addition

## Abstract

The standard addition method is widely used in analytical chemistry to compensate for matrix effects. While effective with single signals (e.g., absorbance at a single wavelength) and independent of matrix composition or blank measurements, it has limitations with high-dimensional data (e.g., full spectra). Existing methods for high-dimensional data require knowledge of the matrix composition and blank measurements, restricting their applicability. We propose a novel algorithm for standard addition that works with high-dimensional data without requiring matrix composition knowledge or blank measurements. By modifying experimental data (e.g., spectra) before applying chemometric models, the algorithm accurately determines analyte concentrations even in complex matrices like seawater or food, where blanks are unavailable. A performance evaluation shows the algorithm compensates effectively for matrix effects, outperforms previously published standard addition algorithms and direct applications of multivariate chemometric algorithms, and is robust to variations in SNR and matrix effect intensity.

## 1. Introduction

Many analytical instruments (e.g., spectrometers) produce signals that are proportional to the analyte concentration. However, additional unknown components can alter the instrument’s sensitivity to the analyte, leading to what is known as the matrix effect. 

This situation frequently arises in environmental analyses, where calibration plots cannot be performed because the composition of the matrix is complex and unknown. A common approach to overcome such matrix effects is the standard addition method. In this method, a calibration plot is constructed by measuring the responses of the original sample after successive additions of known quantities of the analyte. The unknown concentration of the analyte is then determined by extrapolation to the zero-response value (e.g., zero absorbance). Notably, this approach works even when a matrix free of the analyte (blank) is unavailable [1,2].

However, the standard addition method is typically limited to situations where only one signal is recorded per concentration. In contrast, modern instruments can provide multiple signals for each concentration, such as recording an entire absorption spectrum instead of a single wavelength. These systems are termed high-dimensional. When most of the available experimental data are left unused, the analysis may be suboptimal.

A partial solution to this problem has been proposed: build a Partial Least Squares (PLS) calibration model before and after the standard additions. Next, apply this model to the blank signal (collected from a solution containing the same matrix but without the analyte), and predict the analyte concentration via extrapolation [3]. The main drawback is that this approach requires a blank signal, which in turn requires knowledge of the exact matrix composition or a way to prepare the matrix without the analyte. This is usually infeasible in practice because the matrix is often unknown. Consequently, standard addition in high-dimensional data cannot be applied, for example, to compounds in seawater, sludges, or most natural matrices (such as foods or oils).

Despite this obstacle, numerous analytical papers have applied standard addition methods to high-dimensional systems, including spectroscopic [4,5,6,7,8,9,10,11], chromatographic [12,13,14,15,16], sensory [17,18], mass spectrometric [19,20,21], and forensic applications [22,23]. Recently, the standard addition method became useful also in sensor development [24,25,26,27,28,29,30,31,32,33,34,35,36,37,38,39,40,41,42]. In most of these cases, the potential afforded by high dimensionality was not fully utilized, simply because no suitable algorithm was available. Often, since the matrix composition was unknown, most of the measured data could not be used; for example, absorbance at only a single wavelength was utilized, rather than the entire spectrum.

Here, we propose a new standard addition algorithm that enables the use of chemometric regression models (e.g., principal component regression, PCR) in high-dimensional systems. Unlike previous methods, this algorithm remains effective even when the matrix composition is unknown and no blank is available. The direct application of chemometric models (PCR or PLS) alone fails under these conditions, so we propose an additional step. The new algorithm comprises seven straightforward steps, explained below. In essence, we found a way to modify the measured signals (e.g., spectra) before applying the chemometric model, ensuring the correct unknown concentration is recovered. We evaluate the performance of the method as a function of the signal-to-noise ratio (SNR), the magnitude of the matrix effect, and other relevant parameters. The algorithm’s robustness to non-proportionality of the signals is also examined.

## 2. Material and Methods

### 2.1. Data Simulation of Pure Analyte

The algorithm proposed in this study is general; however, we demonstrate its performance and examine its limitations using a specific example. The high-dimensional signal selected for this purpose is a sum of two Gaussian densities, representing typical signals encountered in spectroscopy, such as absorbance as a function of irradiation wavelength. To be specific, the signal at unit concentration of the analyte, without the matrix effect, is given by

(1) $$\varepsilon \left(x_{j}\right)=A_{1}\cdot exp\left(-\frac{{\left(x_{j}-B_{1}\right)}^{2}}{2*25^{2}}\right)+A_{2}\cdot exp\left(-\frac{{\left(x_{j}-B_{2}\right)}^{2}}{2*10^{2}}\right),\;j=1,\ldots ,p.$$

Here, $x_{j}$ is a measurement point. For example, in spectroscopy, *x* is the wavelength. The units of x do not influence the algorithm or its interpretation. The dimension of the signal we explore is p=100. In addition,

$A_{1}$ = 1 (a. u.) represents the first peak height.
$B_{1}$ = 500 is the first peak position.
$A_{2}$ = 1.25⋅A_1_ (a. u.) represents the second peak height.
$B_{2}$ = ${B_{1}+\Delta }_{x}$ is the second peak position.
$\Delta_{x}$ = 70.

**Figure 1:**
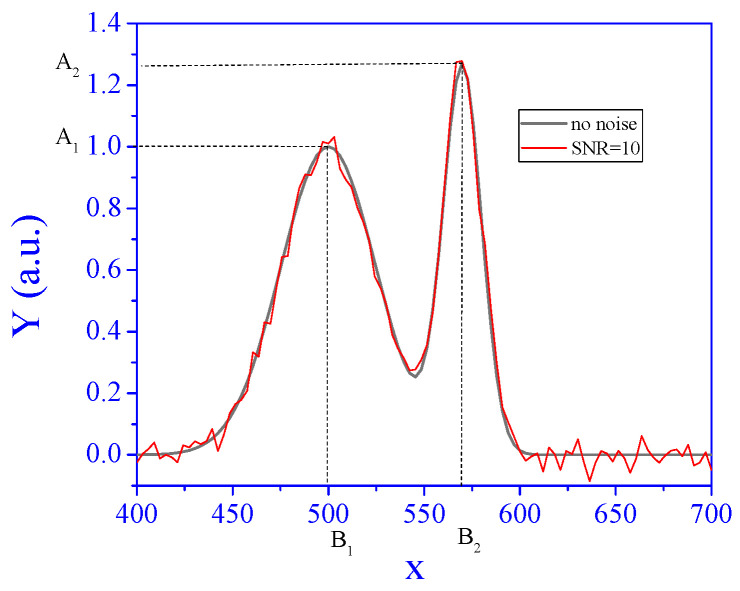
The signal at unit concentration without noise and with SNR of 10.

This signal is shown in Figure 1.

### 2.2. Data Simulation of Noisy Signals

In order to simulate the experimental data, we consider the following statistical measurement error model:

$$f_{i}\left(x_{j}\right)=c_{i}\cdot \varepsilon \left(x_{j}\right),$$

where $f_{i}\left(x_{j}\right)$ is the signal induced by concentration *c_i_* at the zero noise level at each measurement point *x_j_*. $\varepsilon \left(x_{j}\right)$ is the detector response at unit concentration (e.g., molar absorptivity), as defined above in Equation (1). Y, the actually measured signal, including noise, is

$$Y_{ij}=f_{i}(x_{j})+\epsilon_{ij},i=1,...,n,j=1,...,p.$$

The noise $\epsilon_{ij}$ has a Gaussian distribution with expectation 0 and variance σ^2^ for all *i* = 1, …, *n*, *j* = 1, …, *p*. We defined the standard deviation of a signal via a signal-to-noise-ratio (SNR). Let $\mu_{\varepsilon }=\frac{1}{p}\sum_{j=1}^{p}\varepsilon (x_{j})$; then, $\sigma =\frac{\mu_{\varepsilon }}{SNR}$. An example of a noisy signal with SNR = 10 is also shown in Figure 1.

### 2.3. Data Simulation of an Analyte in a Matrix

The matrix affects the sensitivity of the instrument to the analyte concentration, namely changes the proportionality coefficient. We assume that in the presence of the matrix, the signal is

$$f_{i}\left(x_{j}\right)=a\cdot c_{i}\cdot \varepsilon (x_{j}),$$

where *a* represents the degree of the matrix effect (*a* = 1 corresponds to no matrix effect). This parameter can be lower or higher than 1. For simplicity, we use the same notation for the signal with and without matrix, as their usage below will be clear from the context.

### 2.4. Training and Prediction Sets for PCR Analysis

In all simulations, the training sets were of *n* = 50 samples, generated for concentrations from 0.5 to 5.5. The test sets were also composed of 50 samples in the concentration range from 1 to 5.

## 3. Results and Discussion

The goal is to determine the concentration of an analyte in the presence of an unknown matrix for high-dimensional signals. For this purpose, an algorithm (and a program based on it) was developed and tested.

### 3.1. The Algorithm

The algorithm consists of the following steps:

1. Measure a training set of the pure analyte (without matrix effects) at various concentrations. (Include unit concentration and find ε(*x_j_*) at all *j* points, or calculate them from any known concentration.)
2. Create a PCR model for predicting the analyte, based on the above training set.
3. Measure the signals *f*(*x_j_*) at all *j* points of the tested sample (with matrix effects).
4. Add a set of known quantities of the pure analyte to the tested sample, and measure the signals of all the above sets at all points (e.g., wavelengths).
5. For each j=1,…,p, perform a linear regression of the signal vs. added concentration, and note the intercept $\beta_{j}$ and slope $\alpha_{j}.$
6. For each j=1,…,p calculate the corrected signal:
  $$f^{corr}(x_{j})=\varepsilon (x_{j})\frac{\beta_{j}}{\alpha_{j}}$$
7. Apply the PCR model to *f^corr^*, and find the predicted analyte concentration.

**Figure 2:**
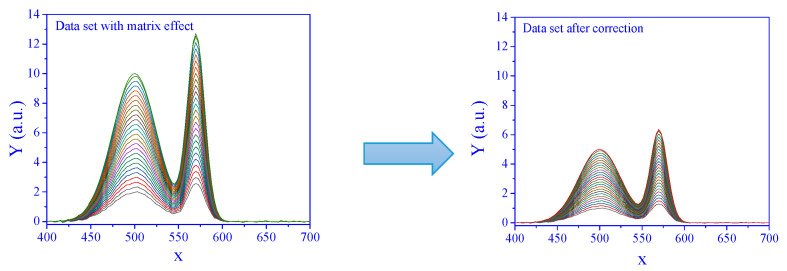
Signal with matrix effect (**left**) and corrected by standard addition (**right**). The degree of the matrix effect, *a*, was 2, and the SNR was 100. The curves correspond to a series of concentrations.

The signals from a set of concentrations subjected to matrix effects, and the corresponding corrected signals are presented in Figure 2.

### 3.2. Testing the Algorithm

Three procedures were performed in order to evaluate the above algorithm. In each of them, the Root Mean Square Error (RMSE) of the prediction was recorded. In all cases, 50 samples of concentrations in the range 1 to 5 were tested, and the RMSE were recorded as a function of the SNR and the degree of the matrix effect:

(a) Testing the predicting ability of the PCR model when no matrix effects are present.
(b) Testing the prediction ability of the PCR model when matrix effects are present (the degree of the matrix effect is different from 1) and the model is directly applied to the measured signals *f*(*x_j_*).
(c) Testing the predictive ability of the PCR model when matrix effects are present, and the model is applied to the corrected signals *f^corr^*.

**Figure 3:**
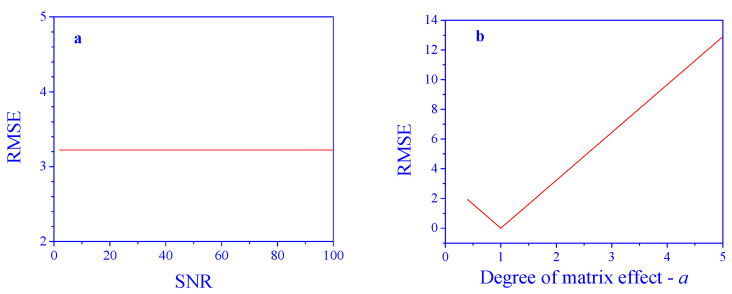
Prediction performance in the presence of matrix effects. (**a**) Effect of SNR (*a* = 2) and (**b**) degree of the matrix effect, *a* (SNR = 10), on concentration prediction error.

As expected, with no matrix effects, the performance of the PCR model is excellent and almost not affected by the SNR in the tested range (RMSE in the order of 10^−15^). But, this is not the case when matrix effects are present. Applying the PCR model to the intact signals results in poor predictions (Figure 3a). Practically, in the studied case, the model is not effective at all. The degree of the matrix effect also has a dramatic influence (Figure 3b), where the RMSE linearly increases as the parameter deviates from 1 in both directions. Even a small deviation makes the model unusable. This demonstrates the necessity of an algorithm to compensate for the matrix effects.

**Figure 4:**
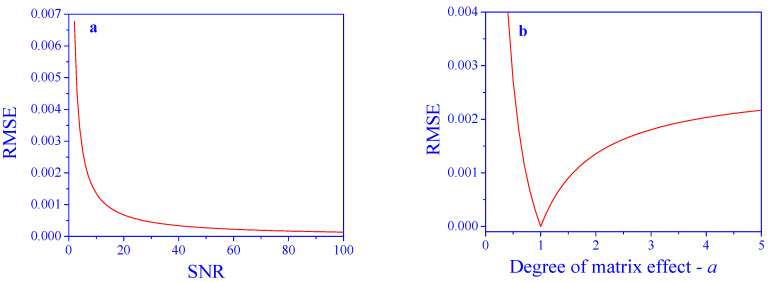
Prediction performance after correcting for matrix effects. (**a**) Effect of SNR (*a* = 2) and (**b**) degree of the matrix effect, a (SNR = 10), on concentration prediction error.

The results of the application of the proposed algorithm are shown in Figure 4. The RMSE decreases as a function of the SNR, as expected. All RMSE values are dramatically improved by the algorithm. For an SNR of 20, the improvement is by a factor of ≈4750, and for an SNR of 40, the improvement is by a factor of ≈9500.

The RMSE values obtained using the proposed algorithm, as a function of the degree of the matrix effect, *a*, are presented in Figure 4b. Also here, all the RMSE values are dramatically improved by the algorithm. At *a* = 1, where there is no matrix effect, the RMSE is practically zero, and it increases in both directions. Nevertheless, the values are rather low and allow for proper analyses in all tested ranges of the parameter. For example, the improvement factor at *a* = 0.5 is ≈590 and at *a* = 4 is ≈4760.

**Figure 5:**
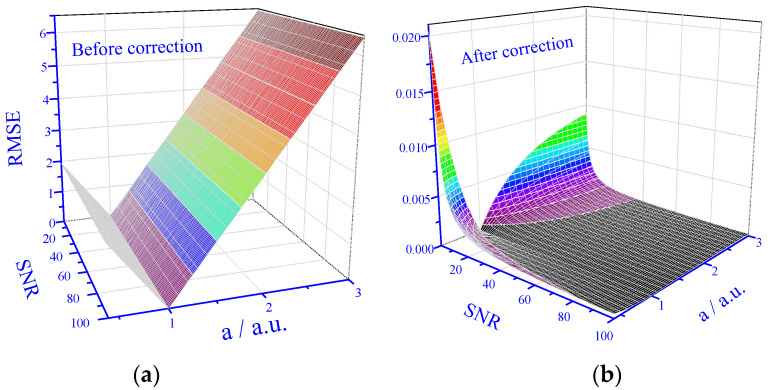
Prediction performance in 3D. (**a**) Prediction performance for data set with matrix effect before correction. (**b**) Prediction performance for data set with matrix effect after correction.

Actually, the RMSE values depend on both the SNR and the degree of matrix effect, *a*. Therefore, 3D maps of the RMSE as a function of both parameters are needed. The results are shown in Figure 5.

The results (Figure 5a) indicate that before application of the correction algorithm, the RMSE dependence on the degree of matrix effect, *a*, is the same for all SNR values in the tested range, and except in the close vicinity of *a* = 1 (no matrix effect), the results are practically unusable. However, after the application of the correction algorithm, the RMSE values dramatically drop (Figure 5b). For low SNR values, the RMSE values strongly increase with deviations from *a* = 1; however, for higher SNR values, there is only little dependence on the degree of the matrix effect and the RMSE values are always very low.

### 3.3. Testing the Sensitivity of the Algorithm to the Signal Shape

The above results were obtained for a specific signal shape, as shown in Figure 1. Here, we analyze the sensitivity of the algorithm to changes in the signal shape. This was carried out by changing the distance between the two peaks, Δx, in the range of −80 to 80.

**Figure 6:**
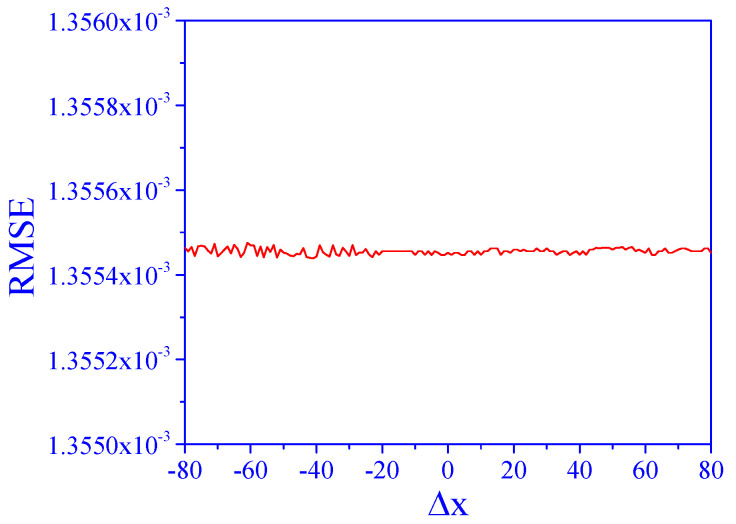
Effect of Δx on concentration prediction, *a* = 2, SNR = 10.

In all tested ranges, direct application of the PCR model to the data (degree of matrix effect of 2 and SNR of 10) resulted in an RMSE above 3, which means total failure of the prediction. Even when the two peaks were well separated, the model failed due to the matrix effects. However, after application of the algorithm, the results (Figure 6) show that the RMSE values were low and practically unchanged in a whole range. This means that the performance of the algorithm is not affected by changing the signal shape in this way.

### 3.4. Testing the Algorithm for Deviations from Proportionality

The PCR model should work well when the signals are linearly dependent on concentrations (but it fails when the matrix effect is present). We have shown that the proposed algorithm, which is based on standard addition, may solve this problem; however, standard addition works only when the signals are proportional to the concentrations. Therefore, it is expected that the performance of the proposed algorithm may deteriorate in the more general, linear case dependence. In order to investigate this degree of deterioration, we tested the effect of adding a constant to the relation between the signal and the concentration:

$$f_{i}\left(x_{j}\right)=a\cdot c_{i}\cdot \varepsilon \left(x_{j}\right)+b$$

where *b* is the additive term.

**Figure 7:**
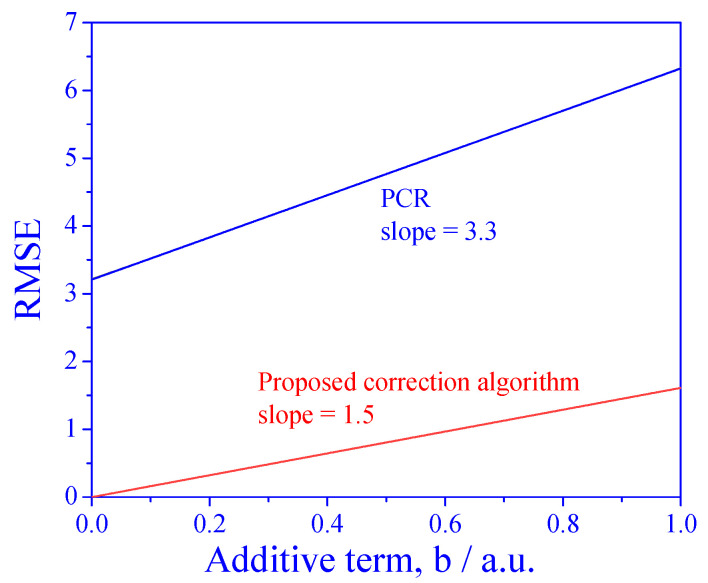
RMSE as a function of deviation from proportionality dependence of the signal on concentration.

The results are shown in Figure 7. The first observation is that for all ranges of the parameter *b*, the RMSE of the proposed algorithm is much better than that of direct PCR. The second observation is that the RMSE increases with the additive term *b* for both the direct application of PCR and for the proposed algorithm. However, the slope of the increase is different: the slope is lower when using the proposed correction algorithm. This means that although standard addition is not supposed to work when *b* is nonzero, the proposed algorithm can handle small deviations from proportionality.

### 3.5. Further Validation Using Monte Carlo Simulations

Furter validation of the results was carried out using Monte Carlo simulations. We ran 1000 simulations with the exact setup described above (focus on the uncertainty of the new observations only) and compared the expected variability with the empirical Monte Carlo variability of the concentration c^*^ = 1.89. The results for SNRs of 10 and 100 are presented in Table 1 and Table 2, correspondingly. Classical and inverse regression estimators were carried out, and both methods provided the same results (as theoretically expected in our case).

The simulations were performed with a considerable matrix effect (of *a* = 2). This should result in a bias of 2c^*^ − c^*^ = 1.89 in our case (see the Theoretical Considerations Section 4 below).

The standard deviation is in agreement with the theoretically predicted value for the data sets without the matrix effect and with the matrix effect. For the corrected data sets, this value is higher, which is due to the additional variance that is coming from randomness of the signals corresponding to the standard additions (note that this additional variance is not taken into account in the theoretical analysis below).

The algorithm works well when the signals are proportional to the concentration of the analyte (e.g., absorbance). Deviations from proportionality dependence might result in inaccurate predictions. This effect is discussed in the next session.

## 4. Theoretical Considerations

### 4.1. The Variance of the Prediction

Recall the measurement error model:

$$Y_{ij}=f_{i}\left(x_{j}\right)+\epsilon_{ij},i=1,...,n,j=1,...,p,$$

where

$$f_{i}\left(x_{j}\right)=c_{i}\cdot \varepsilon \left(x_{j}\right).$$

Let $Y_{i}=\sum_{j=1}^{p}\phi_{j}Y_{ij}$ be some weighted sum of the training observations for all *i* = 1, …, *n*. Then, by applying the inverse regression, we in fact assume for all *i* that *c_i_ ≈ θ*_0_ *+ θ*_1_*Y_i_*. The estimators for *θ*_0_ and *θ*_1_ are easy to obtain as this is just a simple linear regression:

$${\hat{\theta }}_{0}=\bar{c}-{\hat{\theta }}_{1}\bar{Y},\;{\hat{\theta }}_{1}=\frac{\sum_{i=1}^{n}\left(Y_{i}-\bar{Y})(c_{i}-\bar{c}\right)}{\sum_{i=1}^{n}{\left(Y_{i}-\bar{Y}\right)}^{2}},$$

where $\bar{c}=\frac{1}{n}\sum_{i=1}^{n}c_{i}$ and $\bar{Y}=\frac{1}{n}\sum_{i=1}^{n}Y_{i}$.

In particular, $Y_{i}$ are the scores of the first principal component of the training data and $\phi_{j}$ are weights dependent on the $Y_{ij}$s and are given in our case by the estimates of the principal component vector of training data (which we denote by ${\tilde{\phi }}_{j}$). Let ${\tilde{Y}}^{*}=\sum_{j=1}^{p}{\tilde{\phi }}_{j}Y_{j}^{*}$ be a weighted linear combination of a test observation; then, by applying the inverse linear regression (IR) prediction for some c*, we assume that

$$c_{IR}^{*}={\hat{\theta }}_{0}+{\hat{\theta }}_{1}{\tilde{Y}}^{*},$$

where ${\hat{\theta }}_{0}$ and ${\hat{\theta }}_{1}$ are the least square estimates of θ_0_ and θ_1_, respectively, as calculated based on the training data.

Clearly, we would like to understand the performance of such a prediction. The performance will depend on mathematical characteristics of the underlying signal ε, the statistical ones of the error ϵ, and the relations between the two. In the case of the univariate linear relation, the classical least squares estimator is equivalent to the inverse estimator but easier to analyze theoretically. For the classical estimator, we define the following statistical model:

$$Y_{i}=\beta_{0}+\beta_{1}c_{i}+\eta_{i},$$

where $Y_{i}=\sum_{j=1}^{p}\phi_{j}Y_{ij}$, $\beta_{1}=\sum_{j=1}^{p}\phi_{j}\varepsilon_{j}$ and $\eta_{i}=\sum_{j=1}^{p}\phi_{j}\epsilon_{ij}$, *i* = 1, …, n. Note that *β*_0_ will be different from zero when the model is not exact, for example, when we plug in for the weights $\phi_{j}$, their empirical counterpart ${\tilde{\phi }}_{j}$ obtained from estimating the first principal components of the data. A classical regression (CR) prediction is given by

$$c_{CR}^{*}=\frac{{\tilde{Y}}^{*}-{\hat{\beta }}_{0}}{{\hat{\beta }}_{1}}=\frac{\sum_{j=1}^{p}{\tilde{\phi }}_{j}Y_{j}^{*}-{\hat{\beta }}_{0}}{{\hat{\beta }}_{1}},$$

where ${\hat{\beta }}_{0}$ and ${\hat{\beta }}_{1}$ are least square estimates of $\beta_{0}$ and $\beta_{1}$, respectively, obtained from the training data based on the measurement error model defined above.

We would like to understand the performance of such a prediction in terms of their variance and bias in view of several real-life scenarios. We will ignore the bias and variance that may come from the training sample per se for now, meaning that we theoretically calculate and practically simulate the variance and bias conditional on the training sample. We first generate a training data set and obtain the prediction model as explained above and then use a Monte Carlo simulation to assess the testing performance, given the specific training data we obtained.

The estimated $\beta_{1}$ of the classical estimator modeling approach is ${\hat{\beta }}_{1}$ = −3.1019. As mentioned above, $\beta_{1}=\sum_{j=1}^{p}\phi_{j}\varepsilon_{j}$, a quantity we can calculate based on our mathematical model. Let *f* be a *n* × *p* matrix in which the elements are $f_{i}(x_{j})$, *i* = 1, …, *n*, *j* = 1, …, *p*. Let the *p* × *p* matrix $F=f^{T}f$, and denote by $\phi_{j}$, *j* = 1, …, *p* its first eigenvector. The first principal component of the data covariance matrix calculated above is just an estimate of the first eigenvector of *F*. Indeed, such an exact calculation yields $\beta_{1}$ = 3.102.

The variance of the prediction depends on mathematical characteristics of the underlying signal ε, the statistical ones of the error ϵ, and the relations between the two. Let us focus on the uncertainty introduced by the new observation only. In that case, given the training data, the variance of ${\hat{c}}_{CR}^{*}$ comes from the randomness of the test observation only:

$$Var\left[{\hat{c}}_{CR}^{*}\right]=\frac{\sigma^{2}\sum_{j=1}^{p}{\tilde{\phi }}_{j}^{2}}{{\hat{\beta }}_{1}^{2}}=\frac{\sigma^{2}}{{\hat{\beta }}_{1}^{2}}$$

The first equality is because the $Y_{j}^{*}$s are independent for different *j*s, and for all *j*, they have the same variance σ^2^. The second equality is because of the principal components, we have $\sum_{j=1}^{p}{\tilde{\phi }}_{j}^{2}=1$. Specifically, in our setup, we have σ = 0.031, ${\hat{\beta }}_{1}$ = −3.1 so that $\sqrt{Var\left[{\hat{c}}_{CR}^{*}\right]}=0.01$.

### 4.2. The Bias of the Prediction

The expectation of the prediction is given by

$$E\left[{\hat{c}}_{CR}^{*}\right]=\frac{E\left[{\tilde{Y}}^{*}\right]-{\hat{\beta }}_{0}}{{\hat{\beta }}_{1}}=\frac{\sum_{j=1}^{p}{\tilde{\phi }}_{j}E\left[Y_{j}^{*}\right]-{\hat{\beta }}_{0}}{{\hat{\beta }}_{1}}=\frac{c^{*}\sum_{j=1}^{p}{\tilde{\phi }}_{j}\varepsilon_{j}-{\hat{\beta }}_{0}}{{\hat{\beta }}_{1}}$$

Thus, the bias is given by

$$E\left[{\hat{c}}_{CR}^{*}\right]-c^{*}=c^{*}\left(\frac{\sum_{j=1}^{p}{\tilde{\phi }}_{j}\varepsilon_{j}}{{\hat{\beta }}_{1}}-1\right)-\frac{{\hat{\beta }}_{0}}{{\hat{\beta }}_{1}}$$

Note that for a large enough training sample, namely, as *n*→∞, we have ${\hat{\beta }}_{0}\to 0,\;{\hat{\beta }}_{1}\to \beta_{1}$ and $\tilde{\phi }\to \phi$, so that $E\left[{\hat{c}}_{CR}^{*}\right]-c^{*}\to 0$.

Suppose that the test data are coming from a different signal $d_{j}$, as a result of the matrix effect. Then, the test observation is given by

$$Y_{j}^{*}=c^{*}d_{j}+\epsilon_{j},j=1,\ldots ,p.$$

Further, assume that d=a·ε for some positive constant *a*. Then, the expectation of the classical estimator will be

$$E\left[{\hat{c}}_{CR}^{*}\right]=\frac{{a\cdot c}^{*}\sum_{j=1}^{p}{\tilde{\phi }}_{j}\varepsilon_{j}-{\hat{\beta }}_{0}}{{\hat{\beta }}_{1}}\to {a\cdot c}^{*},\;n\to \infty .$$

## 5. Conclusions

We have successfully extended the standard addition method for compensating matrix effects to high-dimensional data in analytical chemistry. Unlike previously reported approaches, the proposed algorithm does not require knowledge of the matrix composition or its characteristics. Instead, it introduces a transformation that modifies the measured signals (e.g., spectra), ensuring accurate concentration estimates when using the PCR model. Our results indicate that the algorithm is robust, showing only minor sensitivity to both the signal-to-noise ratio (SNR) and the magnitude of the matrix effect.

Although the algorithm was exemplified using a particular functional form, our findings suggest that it is only slightly sensitive to this choice and generally outperforms the direct application of PCR. Standard addition inherently relies on signals being proportional to their concentrations; however, our method can accommodate small deviations from strict proportionality. A theoretical study of the variance and bias in concentration predictions aligns closely with the Monte Carlo simulations, further validating the approach. An extension of this methodology to wider multivariate analyses is currently under investigation. The investigation and validation of the suggested algorithm was based solely on simulations. The simulations allowed for extensive and systematic evaluation of performance across a wide range of scenarios, parameters, and conditions, which is often infeasible in laboratory settings [39,40,41,42].

## Figures and Tables

**Table 1:** Results of Monte Carlo simulations (SNR = 10).

	No Matrix	With Matrix	Corrected
mean	1.89	3.78	1.89
std	0.01010	0.00999	0.06016
min	1.86	3.75	1.45
max	1.92	3.81	2.98
bias	−0.0022	1.8876	0.0013

**Table 2:** Results of Monte Carlo simulations (SNR = 100).

	No Matrix	With Matrix	Corrected
mean	1.89	3.78	1.89
std	0.00099	0.00101	0.04927
min	1.89	3.78	0.34
max	1.89	3.78	1.92
bias	−0.0002	1.8898	−0.0019

## Data Availability

No new data were created.
